# Systematic prioritisation of AI-detected chest X-ray abnormalities for optimised lung cancer detection

**DOI:** 10.1093/bjrai/ubag007

**Published:** 2026-03-26

**Authors:** Rhidian R Bramley, Anna C Sharman, Rebecca Duerden, Sarah Lyon, Melissa Ryan, Elodie Weber, Louise Brown, Matthew Evison

**Affiliations:** Greater Manchester Cancer Alliance, c/o The Christie NHS Foundation Trust, Manchester, M20 4BX, United Kingdom; Department of Radiology, Manchester University NHS Foundation Trust, Oxford Road, Manchester, M13 9WL, United Kingdom; Department of Radiology, Stockport NHS Foundation Trust, Stepping Hill Hospital, Poplar Grove, Stockport, SK2 7JE, United Kingdom; Greater Manchester Cancer Alliance, c/o The Christie NHS Foundation Trust, Manchester, M20 4BX, United Kingdom; Annalise.ai , 301/100 Harris Street, Pyrmont, NSW, 2009, Australia; Sectra Imaging IT Solutions, Teknikringen 20, SE-583 30, Linköping, Sweden; Greater Manchester Cancer Alliance, c/o The Christie NHS Foundation Trust, Manchester, M20 4BX, United Kingdom; Department of Radiology, Manchester University NHS Foundation Trust, Oxford Road, Manchester, M13 9WL, United Kingdom; Lung Cancer & Thoracic Surgery Directorate, Wythenshawe Hospital, Manchester University NHS Foundation Trust, Manchester, M13 9WL, United Kingdom; Manchester Academic Health Science Centre (MAHSC), Faculty of Biology, Medicine & Health, The University of Manchester, Manchester, ManchesterUnited Kingdom

**Keywords:** lung cancer, chest X-ray, artificial intelligence, diagnostic triage, radiology workflow, shadow mode, sensitivity, negative predictive value

## Abstract

This paper presents a reproducible, data-driven approach for prioritisation of AI-detected chest X-ray (CXR) findings to support faster lung cancer diagnosis in the NHS. The Annalise Enterprise CXR system was deployed in shadow mode across seven acute trusts in Greater Manchester. Two cohorts were used: a retrospective cancer cohort (*n* = 1,282) with confirmed lung cancer and visible CXR abnormalities, and a prospective cohort (*n* = 13,802) comprising consecutively acquired GP-referred CXRs. Prevalence ratios were calculated for 124 AI-detected abnormalities across both cohorts, and three prioritisation strategies were developed. Strategy 3, which combined prevalence analysis with expert clinical review, achieved optimal performance with a sensitivity of 95.87% and estimated specificity of 79.11%, while maintaining a negative predictive value of 99.95%, for identification of lung cancer. Findings most associated with cancer included solitary lung mass, mediastinal mass, and hilar lymphadenopathy. An Excel-based tool was developed to support rapid configuration and evaluation of categorisation. Application of this approach enabled safe deployment of AI using shadow mode to inform configuration prior to live use. This work provides a scalable model for AI implementation in radiology workflows that aligns with the National Optimal Lung Cancer Pathway and addresses real-world challenges of diagnostic capacity, safety, and reproducibility.

## Introduction

The rapid identification and prompt actioning of imaging, particularly chest x-rays (CXR) that suggest lung cancer, is crucial for improving patient outcomes and aligning with the National Optimal Lung Cancer Pathway.^1^ For most patients, except those presenting with haemoptysis, the first diagnostic test for symptoms that could indicate lung cancer is a CXR.^2^ These symptoms span a range of alternative diagnoses leading to an exceptionally high volume of CXR performed, with over 8 million per year in England.^3^ However, the incidence of lung cancer in CXR requested in primary care is just 1.4%.^4^ This scenario creates substantial demand for CXR reporting in a context where radiology departments are stretched, with most CXR returning normal findings.

According to the 2023 Clinical Radiology Census Report, the NHS spent £276 million on outsourcing, insourcing and temporary locums to manage the overwhelming reporting demand, the highest expenditure on record.^5^ The report also highlighted that this workforce shortages contribute to unacceptable diagnostic delays, including for patients undergoing CXR.

Artificial Intelligence (AI) has been proposed as one solution to these challenges. AI-driven tools can identify CXR findings suspicious for lung cancer and flag them for expedited reporting. Published AI triage approaches have typically relied on expert clinical judgement and have focused on a limited subset of predefined findings, such as pneumothorax or pulmonary nodules, rather than systematic data-driven optimisation.^6–8^

This study addresses the need for a more systematic, data-driven approach to identifying which CXR findings should guide AI prioritisation. The primary objective is to establish a rapid and reproducible method for selecting which AI-detected CXR abnormalities should be used to triage studies for urgent reporting to enable faster diagnosis of lung cancer. Here, we present an approach developed in Greater Manchester that enhances selection through empirical data rather than intuition alone. This study provides robust evidence to guide prioritisation, addressing an important gap in real-world AI implementation.

## Methods

### Study design and population

The study was conducted across seven acute care trusts in Greater Manchester. Two distinct patient cohorts were evaluated with inclusion criteria for each cohort outlined below:

1. **Cohort 1**: A retrospective cohort of 1,282 patients with confirmed lung cancer and an abnormal CXR between 1st June 2020 and 1st June 2022. These cases were curated for a parallel diagnostic accuracy study. Patients were included if they had a CXR within six months of their lung cancer diagnosis, with the CXR report identifying an abnormality that required follow-up.
     Cohort 1 was created by linking the regional lung cancer registry with the radiology archive to identify all patients diagnosed with lung cancer who had undergone a chest X-ray within six months of diagnosis. Each included CXR was classified based on its original radiology report, with inclusion requiring documentation of an abnormality warranting follow up, ensuring representation of both obvious and subtle lesions. Review and classification of radiology reports was performed by a radiology registrar and consultant radiologist, with discrepancies resolved by consensus, in line with the Royal College of Radiologists’ national audit methodology. Cancers that were radiographically occult on chest X-ray, or in which no abnormality was described in the original report, were excluded, as prioritising these would not influence clinical management or reflect the intended function of AI triage.
     The use of this **retrospective, registry-linked cancer cohort**, in combination with a prospective shadow-mode referral cohort, is central to the study design. Shadow-mode evaluation necessarily occurs over a limited period and does not permit long-term follow-up. The registry-linked cancer cohort therefore enables robust estimation of sensitivity and statistical correlation between AI-detected findings and confirmed cancers, while the shadow-mode cohort models their frequency and potential prioritisation effect in routine referrals. This design represents a **real-world, reproducible approach** for pre-deployment AI configuration and safety evaluation.
2. **Cohort 2**: A prospective analysis was designed to include all primary care CXR, performed between 1st June 2024 and 30th July 2024, with the AI system running in shadow mode. All included CXR were GP-referred, collected consecutively and represented the general population undergoing CXR.

For cohort 1, every study was considered positive for lung cancer, owing to the definition of their inclusion criteria. For cohort 2, a 1% detectable lung cancer prevalence was assumed, in line with published studies and previous audits. The assumed prevalence alleviated the need for resource intensive ground truthing and accounted for the low prevalence finding in an otherwise normal cohort.

This study was conducted as a service evaluation under the governance of the participating trusts and did not require formal ethical approval. All data were anonymised prior to analysis in accordance with NHS information governance standards and GDPR regulations. The project complied with the UK Policy Framework for Health and Social Care Research.

### System description and deployment

The **Annalise Container CXR (version 2.1; Annalise-AI)** was deployed using the Sectra Amplifier Platform. This CE-marked and MHRA-registered AI system can detect up to 124 distinct findings on CXR, offering a comprehensive assessment of abnormalities.

The Annalise Enterprise CXR system supports two distinct intended uses:

1. **Clinical decision support**, which generates segmentation overlays and labels on images to assist clinicians by visually indicating the anatomical region associated with each detected abnormality.
2. **AI triage**, which operates entirely in the background to assign a priority level based solely on the binary presence or absence of predefined findings.

This study focused exclusively on the **AI triage** function, which represents a separate intended use under both EU MDR and MHRA CE/UKCA regulatory classifications. Triage operates on the binary presence or absence of findings without using or displaying image overlays. Sites deploying the system are required to define in advance which AI findings should trigger prioritisation for reporting.

Our study was designed specifically to inform this triage configuration step. The objective was not to assess segmentation accuracy, but rather to provide a **statistical correlation** between each AI finding and confirmed lung cancer within a real-world dataset. By comparing finding prevalence between the cancer cohort (Cohort 1) and the general referral population (Cohort 2), the study models what proportion of CXRs in the referral population would be prioritised if particular findings were selected. Because findings can occur in combination, the accompanying Excel tool allows clinical teams to simulate the modelled impact of selecting different findings—quantifying both the proportion of cancers captured and the overall proportion of CXRs that would be prioritised.

During prospective data collection, the AI system was deployed in *shadow mode*, meaning that the outputs were not visible to clinical staff and did not influence patient care or diagnostic decisions. This ensured that the evaluation posed no risks to patient safety while allowing for a robust assessment of the system’s performance. The system analysed every CXR in both cohorts, generating an output for each of the 124 possible findings. The results were then anonymised and exported as comma-separated values (CSV) for further analysis.

Patients were not involved in the design or conduct of this study. As the study focused on evaluating the technical performance of AI in a shadow mode without direct patient impact, patient involvement was not deemed necessary. This study was registered with the Greater Manchester Cancer Research Data Oversight Group as a service evaluation (reference S005).

### AI prioritisation framework development

To support safe and effective AI deployment, we developed a systematic prioritisation framework that identifies which AI-detected chest X-ray (CXR) findings should trigger expedited reporting. The aim was to balance high sensitivity for cancer detection with operational feasibility, using real-world data to inform selection criteria rather than relying solely on clinical intuition.

An Excel-based tool was developed to evaluate the prevalence of AI-detected abnormalities across the two cohorts. The tool imports anonymised CSV exports of AI findings from the cancer and referral cohorts, calculates the prevalence ratio for each finding, and allows users to explore different inclusion thresholds to assess the trade-off between sensitivity and specificity. It also displays the proportion of referral CXRs that would be prioritised under each configuration, helping clinical teams visualise the balance between cancer detection and reporting workload. The tool does not contain patient-identifiable data but requires supplier-specific configuration, as each AI system has a distinct set of detectable findings. Versions of the tool have already been provided to support the two CXR AI suppliers participating in the £21 million National AI Diagnostic Fund (AIDF) deployment, and it is freely available on request for research and NHS use.

The ratio of findings’ prevalence in Cohort 1 compared with Cohort 2 was computed as a key metric to support finding selection. The AI findings were classed into 3 initial priority groups through consensus by the supplier and clinical leads.

1. **CRITICAL—**Findings that potentially require urgent action. These were to be prioritised independent of whether they related to cancer.
2. **HIGH—**Findings of clinical significance that may need to be prioritised.
3. **STANDARD—**Other findings considered not to warrant clinical prioritisation.

Three prioritisation strategies for cancer were developed to determine which of the initial HIGH priority group should be prioritised to optimise the detection of cancer:

1. **Strategy 1**: The 18 AI findings classed as HIGH with a prevalence ratio greater than 5, maximising specificity.
2. **Strategy 2**: All 41 AI findings classed as HIGH, enhancing sensitivity.
3. **Strategy 3**: A final set of 32 AI findings based on clinical judgement, balancing sensitivity and specificity.

A clinical reference group comprising thoracic radiologists, reporting radiographers, and respiratory physicians from the seven NHS Trusts participating in the Greater Manchester AIRPORT study reviewed the findings’ relative prevalence in both cohorts using the Excel tool and refined the final set of findings for prioritisation. This multidisciplinary process balanced statistical association with lung cancer against clinical relevance and workload considerations, producing the final configuration used for Strategy 3.

The Excel-based AI Prioritisation Calculator, containing demonstration data and configuration templates, is available for research and NHS use via GitHub (https://github.com/naidihr/AI_Prioritisation_Tool_Demo).

### Evaluation metrics and statistical analysis

The performance of the AI system was evaluated by calculating *pseudo* sensitivity, specificity, positive predictive value (PPV), negative predictive value (NPV), false positive rate (FPR), and false negative rate (FNR) for each strategy. Sensitivity was determined using only cohort 1 (confirmed lung cancer patients), while specificity, PPV, and NPV were estimated using cohort 2 (general referral population). The Excel tool also calculated the number of CXRs needed to be reported to detect one cancer case to help set expectations for the AI’s clinical impact.

In cohort 1, all chest X-rays (CXRs) were from 1,282 patients with confirmed lung cancer visible on CXR, providing a clear standard to calculate sensitivity:


(*Equation 1*) $$\mathrm{Sensitivity}=\frac{\mathrm{True}\;\mathrm{Positives}\;(TP)}{\mathrm{True}\;\mathrm{Positives}\;(TP)+\mathrm{False}\;\mathrm{Negatives}\;(FN)}$$

For cohort 2, a 1% detectable cancer prevalence was assumed based on published studies and previous audits of primary care CXRs, translating to an estimated 138 cancer positive cases in cohort 2 from a total 13,802 CXR. The True positives in this cohort were estimated as 138 x Sensitivity (computed from Cohort 1). The remaining cancer-negative cases (13,466) minus the false positives (FP) were used to calculate specificity:


(*Equation 2*) $$\mathrm{Specificity}=\;\frac{\mathrm{True}\;\mathrm{Negatives}\;(TN)}{\mathrm{True}\;\mathrm{Negatives}\;(TN)+\mathrm{False}\;\mathrm{Positives}\;(FP)}$$

## Implementation results

**Figure 1:**
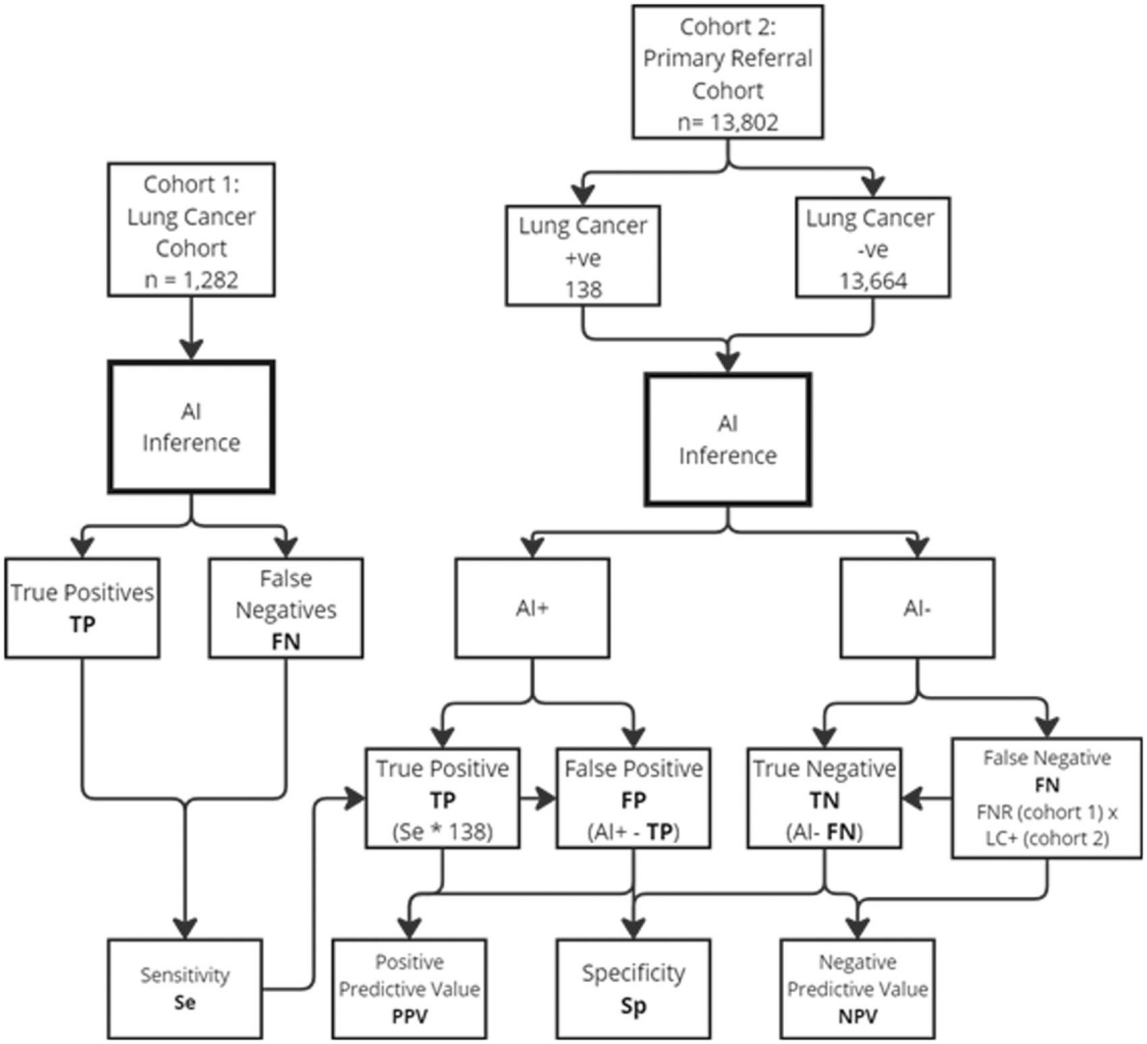
Flow diagram indicating inclusion of studies from each cohort, classification by the AI device and how the performance metrics were computed. The AI classifications varied based on the configuration of findings used.

A total of 1,282 CXRs meeting the inclusion criteria were identified in cohort 1, and 13,802 CXRs meeting the inclusion criteria were included in cohort 2. Based on the assumed 1% lung cancer prevalence in cohort 2, this comprised 138 lung cancer positive cases and 13,664 lung cancer negative cases. The prevalence of all findings in each cohort and relative prevalences are shown in Supplementary Data (ST 1). The mean age in the cancer cohort was 71.4 years (SD ± 9.98), and the mean age in the referral cohort was 59.5 years (SD ± 17.4). There was no difference in the sex ratio between the cancer and referral cohorts, with 46.8% male and 53.2% female in each cohort. The flow of studies from each cohort is illustrated in Figure 1.

The performance of the AI system across the three prioritisation strategies is summarised below and in Table 1:

**Strategy 1**: Prioritising the 18 AI findings classed as HIGH risk with a prevalence ratio greater than 5 for cancer resulted in a sensitivity of 90.80% and specificity of 90.01%. This strategy offered the highest specificity but at the expense of lower sensitivity.
**Strategy 2**: Including AI findings classed as HIGH risk increased sensitivity to 97.27% but reduced specificity to 62.68%.
**Strategy 3**: The final set of findings, incorporating clinical judgement, resulted in a sensitivity of 95.87% in cohort 1, detecting 1,229 of the 1,282 patients with lung cancer. Specificity in cohort 2 was 79.11%, and the NPV was 99.95%.

**Table 1:** Performance metrics of the AI system across three prioritisation strategies for chest X-ray findings, including sensitivity, specificity, positive predictive value (PPV), and negative predictive value (NPV).

Strategy	Strategy 1 (All HIGH >5 ratio)	Strategy 2 (All HIGH)	Strategy 3 (Clinical Review)
**AI Findings selected**	18	41	32
**Percent with AI finding (Cohort 1)**	**90.80%**	**97.27%**	**95.87%**
**Percent with AI finding (Cohort 2)**	**10.80%**	**37.92%**	**21.64%**
True Positive (Cohort 1)	1164	1247	1229
False Negative (Cohort 1)	118	35	53
True Negative (Cohort 2)	12299	8564	10809
False Positive (Cohort 2)	1365	5100	2855
True Positive (Cohort 2)	125	134	132
False Negative (Cohort 2)	13	4	6
**Sensitivity (95% CI)**	90.80% (89.22–92.40%)	97.27% (96.37–98.18%)	95.87% (94.77–96.97%)
**Specificity (95% CI)**	90.01% (89.51–90.52%)	62.68% (61.87–63.49%)	79.11% (78.43–79.79%)
**Positive predictive value (PPV)**	8.41%	2.56%	4.43%
**Negative predictive value (NPV)**	99.90%	99.96%	99.95%
**False Positive Rate (FPR)**	9.99%	37.32%	20.89%
**False Negative Rate (FNR)**	9.20%	2.73%	4.13%
**AI Positive Cancer Detection rate (No CXR to report to detect 1 cancer)**	12	39	23
**AI Negative Cancer Detection rate (No CXR to report to detect 1 cancer)**	969	2274	1895

Percentages for Cohort 2 reflect modelled estimates based on an assumed 1% detectable lung cancer prevalence.

PPV: Positive predictive value–the proportion of AI-positive cases that are true positives.

NPV: Negative predictive value–the proportion of AI-negative cases that are true negatives.

FPR: False positive rate–the proportion of non-cancer cases flagged by AI.

FNR: False negative rate–the proportion of cancer cases missed by AI.

Given that the prevalence of lung cancer within the referral population may vary between sites, we performed a sensitivity analysis, using prioritisation strategy 3, to evaluate the impact of the assumed 1% prevalence on specificity. When varying the assumed lung cancer prevalence rates from 0.5%, to 2% in the referral cohort; the resulting specificities ranged from 78.73% to 79.87%.

Supplementary Figure 1 shows the effect of varying the prevalence ratio threshold when applied across all 124 AI-detected findings, prior to any clinical filtering, illustrating the intrinsic trade-off between sensitivity and the proportion of CXRs prioritised.

### Operational considerations and safety

To ensure patient safety and mitigate risk during the evaluation period, and in alignment with The Royal College of Radiologists’ deployment fundamentals guidance, the AI system was initially deployed in *shadow mode*. In this configuration, AI outputs were not visible to radiologists or clinicians and had no influence on clinical decision-making or patient management. This allowed the performance of the AI system and prioritisation framework to be assessed in a real-world setting without impacting care pathways.

The use of shadow mode ensured that the evaluation could be conducted safely, with no change to existing workflows, enabling trust-level governance teams to approve implementation as a service evaluation. Outputs were reviewed retrospectively, and all data were anonymised in compliance with NHS information governance standards and GDPR.

The study design also avoided any patient-level interventions based on AI outputs, eliminating the risk of automation bias or unintended delays in care. The findings from this shadow mode implementation were used to inform configuration settings, clinical validation, and safety documentation, including preparation of a DCB0160 safety case.

This phased approach, beginning with silent evaluation and supported by quantitative performance metrics, provides a robust and replicable model for the safe integration of AI into radiology workflows.

## Discussion

This study presents a novel and reproducible approach to prioritising CXR AI findings, combining empirical data with clinical judgement to optimise diagnostic accuracy. We employed a two-cohort design to assess both the sensitivity and specificity of the AI system for lung cancer detection on chest X-rays, addressing the challenges of using real-world data in a low-prevalence cancer setting. The cancer prevalence in patients referred from primary care according to NICE NG12 referral guideline in a large retrospective study was 1.4%.^4^ The HealthCare Safety Investigation Branch has flagged that up to 20% of lung cancers are not detectable on CXR,^9^ so the number of detectable cancers is closer to 1%.^10^

Calculating sensitivity required a retrospective cohort (cohort 1) with confirmed lung cancer cases, ensuring a sufficient number of verified positives to serve as the “ground truth”. This approach enabled a rigorous evaluation of the AI system’s ability to detect cancer in a cohort where all cases had a confirmed diagnosis visible on CXR, eliminating the need to wait for sufficient cancer cases to present from the referral population. Importantly, the sensitivity reported in this study relates specifically to lung cancers that were radiographically detectable on chest X-ray at the time of reporting, as defined by contemporaneous radiology reports, rather than to all lung cancers presenting within the diagnostic pathway.

For specificity, we used a prospective cohort (cohort 2) in which the AI was run for a brief period in shadow mode on primary care CXRs. This approach allowed us to estimate specificity and understand the AI’s likely performance in identifying non-cancer cases within a referral population, thereby simulating the AI’s real-world impact without clinical risk.

By combining retrospective sensitivity with prospective prevalence estimates, this study design offered a safe and practical means to inform prioritisation strategies. This two-cohort method provides a valuable framework for assessing AI diagnostic tools prior to live clinical implementation, particularly in areas where the prevalence of the condition in the referral population is low. Clinical judgement was subsequently applied to refine prioritisation strategies, building on this data-driven baseline.

The findings with the highest prevalence ratio in the HIGH priority list included solitary lung mass, inferior mediastinal mass, diffuse upper airspace opacity, diffuse airspace opacity, cavitating mass with content, hilar lymphadenopathy, and multiple masses or nodules. Interestingly, many bone related abnormalities (e.g. kyphosis, osteopaenia, spinal arthritis, and diffuse spinal osteophytes) also exhibited a higher prevalence in the lung cancer cohort. This was attributed to the difference in patient age between the cohorts, the average age in the cancer cohort more than 10 years over the referral cohort. Whilst the prevalence of findings in each cohort provides general insights, the overall objective was to identify a list of findings that would maximise the detection of suspected lung cancer cases, while ensuring critical findings were prioritised, and avoiding the unnecessary prioritisation of low-risk cases (thereby maintaining a high NPV).

### Comparison with other AI triage approaches

It is important to note that the AI system evaluated in this study does not contain a specific finding for “cancer.” Instead, it flags a broad range of radiological abnormalities across the lungs (for example, nodule, mass, collapse, consolidation, interstitial thickening), pleura (for example, effusion or pleural mass), hila, mediastinum, bones, and soft tissues, which may or may not represent malignancy. This evaluation therefore represents a critical step in understanding which AI-detected findings are statistically associated with confirmed lung cancer in real-world practice.

In contrast to other published AI triage methods—often limited to vendor-defined or single-finding prioritisation such as pneumothorax- or nodule-only triage—this study presents a systematic, data-driven method that allows sites to select findings empirically based on their statistical association with cancer and operational impact. This distinction is essential for transparent, reproducible, and regulatorily compliant deployment of AI triage in clinical workflows.

This study demonstrates a systematic approach to selecting AI-detected CXR findings for prioritisation in the context of lung cancer diagnosis. By analysing the prevalence of 124 findings in both a lung cancer cohort and a general referral population, we developed a data-driven method that maximises sensitivity while maintaining a high NPV.

**Strategy 1**, which focused only on the findings with the highest prevalence ratio, achieved high specificity but had a lower sensitivity. **Strategy 2**, which included all non-critical findings that may warrant prioritisation achieved the highest sensitivity but lowest specificity for cancer. **Strategy 3**, which balanced statistical prevalence with clinical judgement, offered the optimal combination of sensitivity and specificity, achieving a sensitivity of 95.87% and an NPV of 99.95%.

While this approach effectively prioritised cases with a high likelihood of cancer, the impact on non-prioritised cases warrants further exploration. Delays in reporting less critical findings could potentially affect patient outcomes for non-cancer conditions. Future studies should evaluate how prioritisation strategies impact overall diagnostic workflows and reporting times for all patients, including those with low-priority findings.

#### Strengths

The approach proposed here differs from traditional methods by implementing a data-driven approach to systematically prioritise findings based on their observed prevalence in cancer versus non-cancer cohorts, rather than relying solely on expert opinion. It is also reproducible across other institutions implementing AI CXR prioritisation using structured AI outputs. The methodology employed here can allow rapid completion of an initial study to support efficient implementation without the need for long term outcome data from prospective cohorts.

The Excel workbook developed as part of this study was instrumental in refining our approach to AI finding prioritisation. By operating on aggregated counts of AI-detected findings and cancer prevalence, the tool can be readily applied to larger populations, different cohort sizes, or an expanded set of AI findings by updating the input data alone, without modification to its underlying structure.

By providing a platform to model different combinations of findings and instantly visualise their impact on diagnostic accuracy, this tool enabled a more data-driven and clinically relevant selection process. Future studies may benefit from adopting similar tools to streamline the integration of AI into clinical practice, particularly in settings where resource optimisation is critical.

### Implementation context

This work represents the shadow-mode and configuration phase that precedes clinical deployment of AI triage systems. Under CE/UKCA and MHRA regulatory frameworks, sites are required to select in advance which of the 124 AI findings will trigger prioritisation. In practice, this involves running the AI system in shadow mode, exporting the outputs for analysis, and using the configuration tool to model the effect of selecting different findings on sensitivity and workload. The agreed configuration is then implemented into the live triage environment following local clinical safety approval (DCB0160). This staged process provides a reproducible and safe pathway for operationalising AI triage within clinical workflows, ensuring that configuration decisions are data-driven and locally validated before live use.

### Clinical safety and real-world assurance

This shadow-mode evaluation also informed the DCB0160 clinical safety case, providing assurance that when AI-based prioritisation is enabled, its effect on reporting order and case triage can be predicted from real-world data. This step ensures that prioritisation does not inadvertently overlook clinically relevant findings and that the benefits of earlier reporting for high-risk cases outweigh any potential delays to non-prioritised examinations. In Manchester, shadow-mode evaluation is followed by post-market monitoring to assess the real-world effects of prioritisation. Together, these processes form a continuous safety and quality cycle, of which this study represents the essential pre-deployment phase.

#### Limitations

The prioritisation system was developed and tested using data cohorts from patients within the same region, which introduces a risk of overestimating clinical performance without external validation. Additionally, the selected findings may not generalise to different geographical areas or populations. Since Strategy 3 was derived using prevalence data from the same cohorts, the reported performance represents calibration rather than independent validation, and real-world performance may differ in external populations. External validation could be achieved by applying the tool to population cohorts outside Greater Manchester, which presents an opportunity for collaboration and further evaluation.

Strategy 3 incorporated clinical judgement through a reference group, which may introduce some degree of human-related biases. However, this input was applied in a constrained and transparent manner on top of predefined data-driven outputs, and the selected findings and decision process are explicitly documented to support reproducibility and local adaptation.

This study did not include retrospective imaging re-review, as its purpose was to configure AI triage for radiographically detectable abnormalities identified at the time of reporting, in line with Royal College of Radiologists audit methodology. It is important to note that the performance metrics reported in this study are indicative rather than definitive. The reference standard for cohort 2 relies on an assumed prevalence, and analyses were conducted at the count level rather than validated against individual study-level reference standards. Consequently, some examinations classified as false positive in Cohort 2 may represent undiagnosed malignancies lacking clinical confirmation within the evaluation period. This would bias specificity estimates downwards, meaning the reported specificity is likely conservative.

Specificity, PPV, and NPV were modelled using an assumed 1% prevalence of detectable lung cancer, based on published UK referral audit data. Because individual patient-level verification was not feasible within the short shadow-mode evaluation period, these estimates should be interpreted as modelled indicators of expected performance rather than definitive diagnostic metrics. The accompanying sensitivity analysis, using prevalence assumptions between 0.5% and 2%, demonstrated that resultant variation in specificity was minimal, supporting the robustness of the modelled estimates.

However, the primary aim of this study was not to determine the diagnostic accuracy of the device but to identify findings that optimise sensitivity and negative predictive value (NPV). Expanding the inclusion of clinically relevant findings and applying the methodology to additional referral populations is expected to influence positive and negative predictive values. Nonetheless, the main objective was to select findings that maximise sensitivity and NPV for identifying suspected lung cancer cases.

Future directions include validating the findings and prioritisation strategies in diverse patient populations and healthcare settings. A priority is to conduct multicentre prospective clinical implementation studies to evaluate real-world performance, including operational impacts on reporting workflows. Collaborations with imaging networks are planned to ensure broader generalisability and external validation of the methodology.

Although patients were not directly involved in the design of this study, future work will incorporate patient perspectives, particularly regarding the ethical implications of prioritisation strategies. Engaging patients in the development of communication strategies for AI prioritisation will enhance transparency and trust in these technologies.

## Conclusion

This study presents a reproducible method for prioritising AI-detected CXR abnormalities, balancing the need for high sensitivity and NPV while accepting a lower PPV to avoid missing cancer cases and ensure early detection. By prioritising findings that maximise sensitivity for radiographically detectable lung cancers on chest X-ray, this approach improves the likelihood of identifying patients with suspected lung cancer. The use of shadow mode ensures clinical safety prior to full deployment, offering a practical, data-driven alternative to traditional judgement-based methods. With further validation, this approach could serve as a blueprint for integrating AI into radiology workflows, offering scalable solutions to global diagnostic challenges.

## Supplementary Material

ubag007_Supplementary_Data

## Clinical relevance statement

The proposed prioritisation approach enhances early lung cancer detection by integrating AI into reporting workflows, addressing radiology demand and workforce constraints.
