# Supplementary material for: Systematic prioritisation of AI-detected chest X-ray abnormalities for optimised lung cancer detection
Source: BJR Artif Intell. 2026 Mar 26;3(1):ubag007. doi: 10.1093/bjrai/ubag007 (PMC13070793; doi:10.1093/bjrai/ubag007)
Supplement: ubag007_Supplementary_Data [file ubag007_supplementary_data.zip › Supplementary Material.docx]

Supplementary data

Supplementary Table 1 (ST1):

| Initial Priority | AI Finding | Final priority | Count of studies (Cohort 1) | Count of studies (Cohort 2) | % of studies (cohort 1) | % of studies (cohort 2) | Prevalence Ratio (Cancer vs Referral) |
| --- | --- | --- | --- | --- | --- | --- | --- |
| Critical | Acute humerus fracture | P1 | 2 | 2 | 0.16% | 0.01% | 10.8 |
| Critical | Acute clavicle fracture | P1 | 1 | 1 | 0.08% | 0.01% | 10.8 |
| Critical | Widened aortic contour | P1 | 3 | 10 | 0.23% | 0.07% | 3.2 |
| Critical | Simple pneumothorax | P1 | 6 | 34 | 0.47% | 0.25% | 1.9 |
| Critical | Acute rib fracture | P1 | 17 | 228 | 1.33% | 1.65% | 0.8 |
| Critical | Pneumomediastinum | P1 | 0 | 1 | 0.00% | 0.01% | 0.0 |
| Critical | Tension pneumothorax | P1 | 0 | 4 | 0.00% | 0.03% | 0.0 |
| Critical | Subdiaphragmatic gas | P1 | 0 | 4 | 0.00% | 0.03% | 0.0 |
| Critical | Suboptimal ETT | P1 | 0 | 0 | 0.00% | 0.00% | 0.0 |
| Critical | Suboptimal NGT | P1 | 0 | 0 | 0.00% | 0.00% | 0.0 |
| Critical | Suboptimal CVC | P1 | 0 | 12 | 0.00% | 0.09% | 0.0 |
| Critical | Scapular fracture | P1 | 0 | 2 | 0.00% | 0.01% | 0.0 |
| Critical | Shoulder dislocation | P1 | 0 | 2 | 0.00% | 0.01% | 0.0 |
| Critical | Suboptimal PAC | P1 | 0 | 0 | 0.00% | 0.00% | 0.0 |
| Critical | Suboptimal gastric band | P1 | 0 | 2 | 0.00% | 0.01% | 0.0 |
| Critical | Subcutaneous emphysema | P1 | 1 | 0 | 0.08% | 0.00% | 0.0 |
| High | Solitary lung mass | P2 | 551 | 72 | 42.98% | 0.52% | 82.4 |
| High | Inferior mediastinal mass | P2 | 91 | 18 | 7.10% | 0.13% | 54.4 |
| High | Diffuse upper airspace opacity | P2 | 23 | 9 | 1.79% | 0.07% | 27.5 |
| High | Diffuse airspace opacity | P2 | 9 | 4 | 0.70% | 0.03% | 24.2 |
| High | Cavitating mass with content | P2 | 30 | 14 | 2.34% | 0.10% | 23.1 |
| High | Spinal wedge fracture | P2 | 18 | 9 | 1.40% | 0.07% | 21.5 |
| High | Hilar lymphadenopathy | P2 | 139 | 74 | 10.84% | 0.54% | 20.2 |
| High | Multiple masses or nodules | P2 | 160 | 87 | 12.48% | 0.63% | 19.8 |
| High | Perihilar airspace opacity | P2 | 13 | 8 | 1.01% | 0.06% | 17.5 |
| High | Lung collapse | P2 | 12 | 8 | 0.94% | 0.06% | 16.1 |
| High | Cavitating mass(es) | P2 | 64 | 53 | 4.99% | 0.38% | 13.0 |
| High | Diffuse lower airspace opacity | P2 | 54 | 55 | 4.21% | 0.40% | 10.6 |
| High | Solitary lung nodule | P2 | 411 | 474 | 32.06% | 3.43% | 9.3 |
| High | Segmental collapse | P2 | 181 | 210 | 14.12% | 1.52% | 9.3 |
| High | Loculated effusion | P2 | 31 | 39 | 2.42% | 0.28% | 8.6 |
| High | Focal airspace opacity | P2 | 639 | 825 | 49.84% | 5.98% | 8.3 |
| High | Multifocal airspace opacity | P2 | 104 | 144 | 8.11% | 1.04% | 7.8 |
| High | Spinal lesion | P2 | 1 | 2 | 0.08% | 0.01% | 5.4 |
| High | Pleural mass | P2 | 37 | 83 | 2.89% | 0.60% | 4.8 |
| High | Superior mediastinal mass | P2 | 56 | 131 | 4.37% | 0.95% | 4.6 |
| High | Diffuse pleural thickening | P2 | 15 | 37 | 1.17% | 0.27% | 4.4 |
| High | Upper interstitial thickening | P2 | 195 | 508 | 15.21% | 3.68% | 4.1 |
| High | Upper zone fibrotic volume loss | P2 | 53 | 157 | 4.13% | 1.14% | 3.6 |
| High | Rib lesion | P2 | 39 | 122 | 3.04% | 0.88% | 3.4 |
| High | Simple effusion | P2 | 289 | 953 | 22.54% | 6.90% | 3.3 |
| High | Diffuse nodular / miliary lesions | P2 | 20 | 68 | 1.56% | 0.49% | 3.2 |
| High | Lower zone fibrotic volume loss | P2 | 24 | 89 | 1.87% | 0.64% | 2.9 |
| High | Tracheal deviation | P2 | 75 | 285 | 5.85% | 2.06% | 2.8 |
| High | Diffuse interstitial thickening | P2 | 57 | 292 | 4.45% | 2.12% | 2.1 |
| High | Scapular lesion | P2 | 2 | 12 | 0.16% | 0.09% | 1.8 |
| High | Humeral lesion | P2 | 6 | 51 | 0.47% | 0.37% | 1.3 |
| High | Internal foreign body | P2 | 1 | 38 | 0.08% | 0.28% | 0.3 |
| High | Clavicle lesion | P2 | 0 | 9 | 0.00% | 0.07% | 0.0 |
| High | Calcified mass (> 5mm) | P3 | 23 | 126 | 1.79% | 0.91% | 2.0 |
| High | Pulmonary artery enlargement | P3 | 13 | 79 | 1.01% | 0.57% | 1.8 |
| High | Basal interstitial thickening | P3 | 169 | 1042 | 13.18% | 7.55% | 1.7 |
| High | Distended bowel | P3 | 5 | 50 | 0.39% | 0.36% | 1.1 |
| High | Widened cardiac silhouette | P3 | 170 | 2080 | 13.26% | 15.07% | 0.9 |
| High | Diffuse fibrotic volume loss | P3 | 2 | 41 | 0.16% | 0.30% | 0.5 |
| High | Pulmonary congestion | P3 | 16 | 333 | 1.25% | 2.41% | 0.5 |
| High | Peribronchial cuffing | P3 | 26 | 814 | 2.03% | 5.90% | 0.3 |
| Standard | Kyphosis | P3 | 23 | 6 | 1.79% | 0.04% | 41.3 |
| Standard | Osteopaenia | P3 | 21 | 8 | 1.64% | 0.06% | 28.3 |
| Standard | Spinal arthritis | P3 | 42 | 18 | 3.28% | 0.13% | 25.1 |
| Standard | Diffuse spinal osteophytes | P3 | 4 | 2 | 0.31% | 0.01% | 21.5 |
| Standard | Post resection volume loss | P3 | 19 | 60 | 1.48% | 0.43% | 3.4 |
| Standard | Lower zone bullae | P3 | 5 | 16 | 0.39% | 0.12% | 3.4 |
| Standard | Upper zone bullae | P3 | 27 | 88 | 2.11% | 0.64% | 3.3 |
| Standard | Rib resection | P3 | 3 | 12 | 0.23% | 0.09% | 2.7 |
| Standard | Reduced lung markings | P3 | 45 | 191 | 3.51% | 1.38% | 2.5 |
| Standard | Aortic stent | P3 | 2 | 10 | 0.16% | 0.07% | 2.2 |
| Standard | Diffuse bullae | P3 | 1 | 5 | 0.08% | 0.04% | 2.2 |
| Standard | Aortic arch calcification | P3 | 539 | 3016 | 42.04% | 21.85% | 1.9 |
| Standard | Gallstones | P3 | 3 | 17 | 0.23% | 0.12% | 1.9 |
| Standard | Calcified hilar lymphadenopathy | P3 | 3 | 17 | 0.23% | 0.12% | 1.9 |
| Standard | Hyperinflation | P3 | 318 | 1938 | 24.80% | 14.04% | 1.8 |
| Standard | Mastectomy | P3 | 25 | 158 | 1.95% | 1.14% | 1.7 |
| Standard | Coronary stent | P3 | 9 | 59 | 0.70% | 0.43% | 1.6 |
| Standard | Incompletely imaged chest | P3 | 108 | 717 | 8.42% | 5.19% | 1.6 |
| Standard | Calcified pleural plaques | P3 | 34 | 226 | 2.65% | 1.64% | 1.6 |
| Standard | Diaphragmatic elevation | P3 | 138 | 950 | 10.76% | 6.88% | 1.6 |
| Standard | Calcified axillary nodes | P3 | 5 | 35 | 0.39% | 0.25% | 1.5 |
| Standard | Shoulder replacement | P3 | 7 | 50 | 0.55% | 0.36% | 1.5 |
| Standard | Mediastinal clips | P3 | 42 | 305 | 3.28% | 2.21% | 1.5 |
| Standard | Axillary clips | P3 | 21 | 169 | 1.64% | 1.22% | 1.3 |
| Standard | Patient rotation | P3 | 305 | 2509 | 23.79% | 18.18% | 1.3 |
| Standard | Bronchiectasis | P3 | 25 | 209 | 1.95% | 1.51% | 1.3 |
| Standard | Hiatus hernia | P3 | 34 | 298 | 2.65% | 2.16% | 1.2 |
| Standard | Lung sutures | P3 | 4 | 37 | 0.31% | 0.27% | 1.2 |
| Standard | Atelectasis | P3 | 162 | 1529 | 12.64% | 11.08% | 1.1 |
| Standard | Electronic cardiac devices | P3 | 29 | 274 | 2.26% | 1.99% | 1.1 |
| Standard | Calcified granuloma (< 5mm) | P3 | 24 | 235 | 1.87% | 1.70% | 1.1 |
| Standard | Chronic rib fracture | P3 | 48 | 474 | 3.74% | 3.43% | 1.1 |
| Standard | Unfolded aorta | P3 | 321 | 3333 | 25.04% | 24.15% | 1.0 |
| Standard | Scoliosis | P3 | 74 | 810 | 5.77% | 5.87% | 1.0 |
| Standard | Chronic humerus fracture | P3 | 5 | 56 | 0.39% | 0.41% | 1.0 |
| Standard | Rotator cuff anchor | P3 | 7 | 81 | 0.55% | 0.59% | 0.9 |
| Standard | Sternotomy wires | P3 | 30 | 349 | 2.34% | 2.53% | 0.9 |
| Standard | Gastric band | P3 | 1 | 12 | 0.08% | 0.09% | 0.9 |
| Standard | Diaphragmatic eventration | P3 | 107 | 1310 | 8.35% | 9.49% | 0.9 |
| Standard | Breast implant | P3 | 5 | 62 | 0.39% | 0.45% | 0.9 |
| Standard | Nipple shadow | P3 | 24 | 301 | 1.87% | 2.18% | 0.9 |
| Standard | Chronic clavicle fracture | P3 | 8 | 102 | 0.62% | 0.74% | 0.8 |
| Standard | Shoulder fixation | P3 | 2 | 26 | 0.16% | 0.19% | 0.8 |
| Standard | Underexposed | P3 | 2 | 30 | 0.16% | 0.22% | 0.7 |
| Standard | Abdominal clips | P3 | 23 | 368 | 1.79% | 2.67% | 0.7 |
| Standard | Cervical flexion | P3 | 5 | 80 | 0.39% | 0.58% | 0.7 |
| Standard | Neck clips | P3 | 4 | 65 | 0.31% | 0.47% | 0.7 |
| Standard | Shoulder arthritis | P3 | 5 | 82 | 0.39% | 0.59% | 0.7 |
| Standard | Spinal fixation | P3 | 2 | 35 | 0.16% | 0.25% | 0.6 |
| Standard | Pericardial fat pad | P3 | 11 | 368 | 0.86% | 2.67% | 0.3 |
| Standard | Cardiac valve prosthesis | P3 | 2 | 88 | 0.16% | 0.64% | 0.2 |
| Standard | Underinflation | P3 | 1 | 90 | 0.08% | 0.65% | 0.1 |
| Standard | Airway stent | P3 | 0 | 1 | 0.00% | 0.01% | 0.0 |
| Standard | Clavicle fixation | P3 | 0 | 17 | 0.00% | 0.12% | 0.0 |
| Standard | Calcified neck nodes | P3 | 0 | 13 | 0.00% | 0.09% | 0.0 |
| Standard | Image obscured | P3 | 0 | 0 | 0.00% | 0.00% | 0.0 |
| Standard | Intercostal drain | P3 | 0 | 0 | 0.00% | 0.00% | 0.0 |
| Standard | In position CVC | P3 | 0 | 18 | 0.00% | 0.13% | 0.0 |
| Standard | In position NGT | P3 | 0 | 0 | 0.00% | 0.00% | 0.0 |
| Standard | In position ETT | P3 | 0 | 0 | 0.00% | 0.00% | 0.0 |
| Standard | Biliary stent | P3 | 0 | 3 | 0.00% | 0.02% | 0.0 |
| Standard | Oesophageal stent | P3 | 0 | 1 | 0.00% | 0.01% | 0.0 |
| Standard | In position PAC | P3 | 0 | 0 | 0.00% | 0.00% | 0.0 |
| Standard | Rib fixation | P3 | 0 | 2 | 0.00% | 0.01% | 0.0 |
| Standard | Pectus excavatum | P3 | 0 | 2 | 0.00% | 0.01% | 0.0 |
| Standard | Overexposed | P3 | 0 | 0 | 0.00% | 0.00% | 0.0 |
| Standard | Pectus carinatum | P3 | 0 | 0 | 0.00% | 0.00% | 0.0 |
| No Findings | NULL - no findings | P3 | 4 | 3350 | 0.31% | 24.27% | 0.0 |

Supplementary Figure 1 (SF1):

[File uploaded separately as per author instructions]

**Supplementary Figure 1.** Effect of varying prevalence-ratio thresholds on sensitivity for radiographically detectable lung cancer (Cohort 1) and the proportion of CXRs prioritised (Cohort 2), restricted to AI-detected findings classified as HIGH priority. The figure illustrates the trade-off between cancer detection sensitivity and reporting workload prior to the application of clinical refinement.
