# Supplementary figures and images for: Systematic prioritisation of AI-detected chest X-ray abnormalities for optimised lung cancer detection
Source: BJR Artif Intell. 2026 Mar 26;3(1):ubag007. doi: 10.1093/bjrai/ubag007 (PMC13070793; doi:10.1093/bjrai/ubag007)

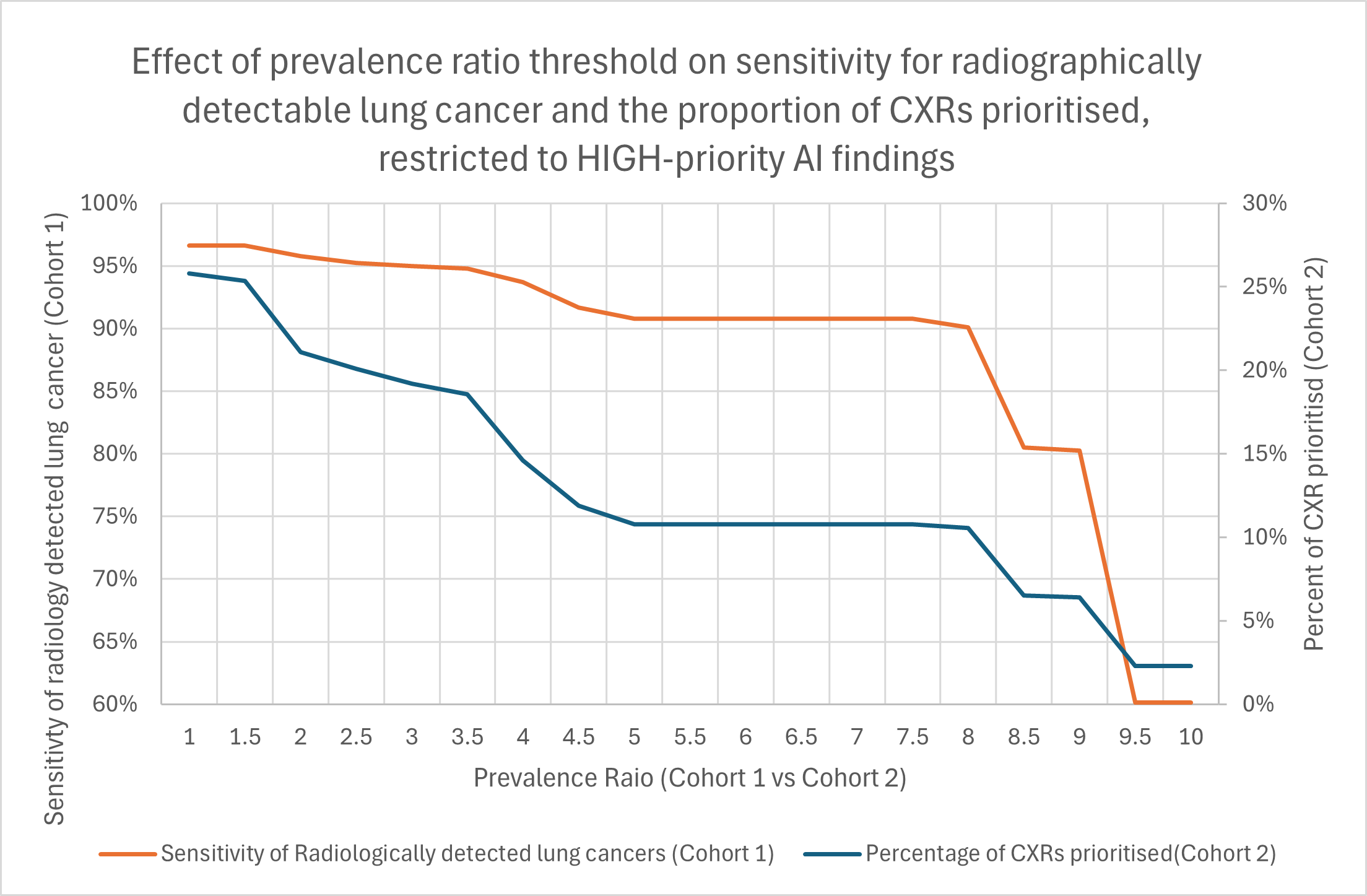

Supplement: ubag007_Supplementary_Data [file ubag007_supplementary_data.zip › Supplementary Figure 1.png]
